# The Embodied and Situated Nature of Moods

**DOI:** 10.1007/s11406-017-9817-0

**Published:** 2017-02-13

**Authors:** Giovanna Colombetti

**Affiliations:** Department of Sociology, Philosophy and Anthropology, University of Exeter, Exeter, UK

**Keywords:** Moods, Embodiment, Situated cognition, Incorporation, Scaffolded cognition

## Abstract

In this paper I argue that it is misleading to regard the brain as *the* physical basis or “core machinery” of moods. First, empirical evidence shows that brain activity not only influences, but is in turn influenced by, physical activity taking place in other parts of the organism (such as the endocrine and immune systems). It is therefore not clear why the core machinery of moods ought to be restricted to the brain. I propose, instead, that moods should be conceived as *embodied*, i.e., their physical basis should be enlarged so as to comprise not just brain but also bodily processes. Second, I emphasise that moods are also *situated* in the world. By this I do not simply mean that moods are influenced by the world, but that they are complexly interrelated with it, in at least three different ways: they are shaped by cultural values and norms; they are materially and intersubjectively “scaffolded”; and they can even “experientially incorporate” parts of the world, i.e., include the experience of parts of the world as part of oneself.

## Introduction

There is a widespread tendency, in affective neuroscience, to regard the brain as *the* physical basis or “core machinery” of moods and other affective states (emotions, pain, pleasure) and, relatedly, to assume that an explanation of the workings of the brain will provide a complete explanation of the physical underpinnings of those states. Of course, neuroscientists are by definition scientists of the brain; it is their job to figure out how the brain works and, in the case of affective neuroscience, to discover how the brain operates during affective episodes. It seems also undeniable that the brain, in those organisms that possess one, is necessary for moods. Yet all too often the message from neuroscience is that the brain is the privileged site, or the *locus*, of affective phenomena, and that physical processes taking place outside the brain are mere “background conditions”.^1^

In this paper I advance a variety of considerations aimed at challenging this brain-bound or “embrained” view of affective states. Given the theme of this special issue, I focus on moods, and specifically on human moods.^2^ Like many philosophers, I take moods to be different from emotions because of their different way of being intentionally related to the world. Moods such as depression, anxiety, irritation, elation, optimism, or simply “being up” or “being down” are not about anything in particular (see, e.g., Broad 1954; Lormand 1985; Ben-Ze’ev 2010). Emotions, on the other hand, are typically about specific objects, people or events: I am angry at my neighbour, afraid of the snake, and worried about the results of the referendum.

My aim in this paper is to argue that moods are *embodied* and *situated*. I do not deny that brain activity plays a very important, even necessary, role in moods. I argue, however, that the physical basis of moods should not be restricted to the brain (or to some specific part of it). Rather, in my view, moods are best regarded as embodied (section 2), in the sense that their physical basis extends beyond the brain, to include processes going on in other parts of the organism. In addition, I emphasize that moods are situated in the world (section 3). This claim is, I believe, uncontroversial, but it needs to be spelled out in more detail, introducing conceptual distinctions to capture different modalities of situatedness. I suggest that moods are situated not just in the sense that they are influenced by events and situations in the world in a relatively simple linear way; rather, they are complexly interrelated with the world, in at least three different ways: i) they are shaped by cultural norms and values, ii) they are “scaffolded” by objects and people, and iii) they can even “experientially incorporate” parts of the world, i.e., include the experience of those parts as part of oneself.

## The Embodiment of Moods

Research in the neurobiology of moods is ongoing, but there is little doubt that our moods depend on physical processes going on in our brain. For example, we know from neuroscientific and pharmacological research on mood disorders (mainly depression and anxiety) that changes in neurotransmitters and neuromodulators come with changes in mood experiences and mood-related behaviour.^3^ For example, dopamine, serotonin and noradrenaline appear to play a role in keeping our mood elevated, and they are all present in significantly lower levels in depressed individuals (e.g., Diehl and Gershon 1992; Young and Leyton 2002; Palazidou 2012; Singh 2014; Jenkins et al. 2016).

Importantly, however, one should not conclude from this kind of findings that these neurotransmitters and neuromodulators, together with the neurons with which they interact, constitute *the* physical basis or “core machinery” of moods, and that a complete explanation of these brain processes will provide a complete explanation of the physical mechanisms underpinning moods. This is because neurotransmitters and neuromodulators operating within the skull are not like liquids in a sealed jar; they interact with other parts of the organism, and moreover this interaction influences our moods. For example, chemical activity in the brain is influenced by rates of blood glucose in the bloodstream (e.g., Koshimura et al. 2003), and it has been found that both hypoglycaemia and hyperglycaemia have detrimental effects on mood (e.g., Gold et al. 1995; Sommerfield et al. 2004). Brain activity is also influenced by a variety of hormones (Nelson 2011). Hormones, it is worth emphasizing, are chemical substances released into the bloodstream by endocrine glands located in *both* brain and body, such as the pituitary gland or hypophysis (located in the base of the skull), the adrenal glands (located above the kidneys), and the ovaries and testes. Importantly, hormones are peptides just like neurotransmitters and neuromodulators, and often the same type of peptide will act as a neurotransmitter, as a neuromodulator, and as a hormone. Noradrenaline, for example, acts as a neurotransmitter in the brain, but also as a hormone produced by the adrenal glands (Widmaier et al. 2014). In fact, many of the mood-influencing peptides that act as neurotransmitters and neuromodulators in the brain are produced outside the brain; significantly, 95% of serotonin is synthesised in the gastrointestinal tract (O’Mahony et al. 2015; Yano et al. 2015; Jenkins et al. 2016); similarly for dopamine, which is also produced in the gut and, in smaller quantities, in the adrenal medulla (Eisenhofer et al. 1997).

In addition, the nervous system (central and peripheral) interacts with the immune system. Until the 1970s, these two systems were thought to be independent and distinct, with the immune system having its own mechanisms of communication and even memory (Bottaccioli 2008). Since then, however, we know that the two systems influence each other, and that their interactions induce fluctuations in mood. Notable here is the recent discovery that innate immune cells in the body can produce pro-inflammatory proteins, known as cytokines, that reach the brain and influence neuronal activity, and whose production during illness causes states of fatigue and depressed mood (Dantzer et al. 2008).

Perhaps the mood with the best understood physiological basis is stress. The stress response is said to involve both a “neural” and a “chemical” path (Bottaccioli 2005, 193–195; Charmandari et al. 2005; Smith & Vale 2006). On the one hand, the hypothalamus responds to the presence of a threat by stimulating the sympathetic nervous system to produce excitatory substances, such as adrenaline, noradrenaline and dopamine (neural path). On the other hand, the hypothalamus also releases CRH (corticotropin-releasing hormone) into the bloodstream. CRH acts as a neurotransmitter in the brain, and also stimulates the pituitary to release ACTH (adrenocorticotropic hormone), which in turn stimulates the adrenal glands to release another hormone, cortisol (chemical path). These processes occur at different timescales. Whereas the sympathetic nervous system is excited shortly after the initial hypothalamic response, ACTH and cortisol are released later, with cortisol elevation occurring after 15 to 30 min. At this stage, if the threat subsides, cortisol suppresses the hypothalamus and pituitary responses, including the release of CRH and ACTH, and the stress response subsides as well (cortisol thus regulates its own secretion via a negative feedback loop). If the threat continues, the stress response continues too, with cortisol contributing to setting up a physiological long-term stress response.

This account clearly does not reduce the physical basis of stress to the brain, because it also describes the important role of chemical activity going on in (specific parts of) the body in constituting the organism’s stress response. Moreover, the account describes neural and chemical processes that influence one another. There thus appear to be no good reasons for restricting the physical basis of stress to the brain, or to a specific part of it (such as, say, the hypothalamus, the hypothalamic-pituitary axis, or any other pattern of brain activity going on during a stress response). Given the apparent necessary role of neural and chemical activity taking place in the body to sustain a stress response, and the mutual influences occurring between brain and body, the more appropriate view, I maintain, is the one that regards the physical basis of stress as comprising a brain-body neuroendocrine system—one that includes neurochemical processes in the brain but also the release of chemical substances synthetized locally in the body.

In light of this evidence, someone who reiterated that only activity taking place in the brain counts as the physical basis of stress (or that “stress is in the brain”), and that activity taking place in the body just provides background conditions (perhaps it only “modulates” or “moderates” brain-based stress), would need to answer the following question: why, then, not regard just a small set of neurons, or perhaps only one neuron (in the hypothalamus, say), as the physical basis of stress, and the other brain (and body) areas involved in the stress response as background or modulating conditions? If one is willing to characterize neurochemical patterns of looping processes in the brain as constituting the physical basis of a certain affective condition (as most neuroscientists already do), the fact that these loops go well beyond the brain and involve chemical processes taking place in the body should count as a good reason for not confining such physical basis to the brain only.^4^

One question at this juncture is, of course, whether *all* moods, including everyday ones such as elation, grumpiness or irritability, can be said to be underpinned by physical systems involving both brain and bodily processes. Admittedly, whereas the neurochemistry of the stress system and of mood disorders like depression has been paid quite a lot of attention, we know much less about the physiology of these other, less clinically relevant affective states. Yet, given what we know about how the brain and the rest of the organism continuously influence one another (see Thompson and Cosmelli 2012), it is plausible to hypothesize that brain-body continuous interactions of various kinds will affect our everyday moods too, and thus that not just clinically relevant mood disorders, but everyday moods as well, are embodied. More empirical evidence is needed, however, to identify the specific brain-body neurochemical bases of these states.

## Moods in the World

Let us turn now to the situated character of moods. As embodied beings, we do not float about in a vacuum, but we interact with the world and with the animate and inanimate entities that are in it; relatedly, our moods do not depend only on the state of our organism, but also on our interactions with the world. This is all fairly trivial, although surprisingly easy to overlook. In particular, there is a tendency in affective science to oversimplify or even forget the complex role of the environment. The latter is typically reduced to a set of stimuli that bring about, in a relatively simple linear way, a set of mood responses in the organism. As Griffiths & Scarantino (2009, p. 437) have pointed out, in the most influential theories of emotions, “emotions are conceived as internal states or processes and the role of the environment is confined to providing stimuli and receiving actions”. The same consideration applies to moods as well.

In this section I show that the relation between environment and moods is much more than a simple stimulus-response link. Moods are situated in the sense of complexly interrelated with various aspects of the world. Drawing from cultural psychology, I show that the world provides not just stimuli, but also cultural values, norms, and rules of behaviour that shape and impact moods at various levels (section 3.1). In addition, we do not just passively “respond” to the world, but also *actively* modify it in order to influence our moods, becoming able to undergo experiences that would otherwise be out of our reach. Borrowing an increasingly popular term from the philosophy of situated cognition, I propose that the world *scaffolds* our minds—not just our cognitive capacities, but also our affective ones (section 3.2). Finally, drawing partly on phenomenological philosophy, I suggest that moods can *experientially incorporate* parts of the world, namely, part of the experience of undergoing a mood may include experiencing certain aspects of the world as part of oneself (section 3.3).

### Cultural Influences

Our encounters with objects and people in the world affect our moods, but the world influences our moods in more indirect ways as well. As cultural psychologists have shown, cultural values and norms influence how we feel and behave in ways we are often not aware of (Mesquita et al. 2016).

Most cultural-psychological studies have looked at specific emotions, but some of their results apply to moods as well. Oishi et al. (2004), for example, showed that the mood of Japanese and Hispanics is more dependent on their social situation (i.e., whether they are with friends, relatives, spouse, co-workers, etc.) than the mood of North Americans. Others have found that “ideal affect” (the affective experiences people value and want to have; see, e.g., Tsai 2007) differs across cultures, and that these differences correlate with differences in people’s mood. For example, North Americans appear to value intense, high-arousal positive moods (such as excitement) more than Hong Kong Chinese, who instead appear to favour calmer or less aroused positive moods (such as contentment). Relatedly, North Americans seek states of excitement more often than Hong Kong Chinese, and tend to be comparatively more depressed when they do not achieve them; conversely for Hong Kong Chinese (Tsai et al. 2006).

The influence of cultural values is apparent also in the finding that people from different cultures appear to differ in how they *regulate* their moods. “Mood self-regulation” is an important construct in affective science and particularly clinical psychology, where it refers to the strategies individuals employ to boost, maintain, or reduce their current mood (e.g., Thayer 1996; Larsen 2000).^5^ Culture appears to influence mood self-regulation by influencing how people evaluate being in a certain mood, notably whether they regard a certain mood as positive or not, and thus whether they want to get rid of it or not. Thus, North Americans appear to regulate anger (understood not just as an emotion but also as a mood, such as general irritation and grumpiness) differently from Tibetans. Whereas North Americans do not always repress or try to control their anger, Tibetans have developed various practices, such as compassion-based meditations, to control and deflate it, and to reduce its duration and intensity. This is likely because North Americans, as indicated by self-reports, have an ambivalent relation toward anger: they regard it as a natural response to offense or unfairness that can be harmful to the angry individual and to the people around her, but that can also motivate acting and helping others. Tibetans, on the other hand, regard anger (*lung lang*) as always unwholesome and harmful to the angry person and those around her, and consider being able not to be overcome by anger a sign of wisdom and higher spiritual achievement (Shweder et al. 2008).

Finally, there is also some evidence that culture influences how moods are behaviourally manifested, although it is harder to come by. Research on facial expressions has studied primarily emotions rather than moods, and has looked for pancultural commonalities rather than cross-cultural differences (e.g., Ekman 2003). One exception is Elfenbein et al. (2007), who found differences in the way participants from Gabon and Quebec express mood-like affective states of serenity, anger and sadness in the face. Discussions of cultural variations in body language, even if often based on personal observations rather than rigorous ethological research, are also relevant here. Morris (1977/2002), for one, provides a number of interesting descriptions (even though some are a bit out-dated). Consider for example the *moutza*—a Greek gesture involving thrusting one’s open hand with widely spread fingers toward someone else’s face. This gesture is highly insulting in Greece, and is thus presumably performed by someone in a negative mood involving irritation and feeling offended. Or consider the sign performed by joining together the thumb and the index finger to make a circle. This gesture has different meanings in different cultures: in North America it is an “OK” sign that indicates things are going well, in Japan apparently it means “money”, in parts of France it means “zero” or “worthless”, in Malta it refers derogatorily to someone being a (male) homosexual, and in Sardinia and Greece it is an obscene insult with sexual connotations. We can then plausibly think of this gesture as being performed as part of the bodily manifestations of a mood, although a different mood in each of these countries: a North American may perform the gesture to manifest an optimistic mood, a French may perform it as part of a pessimistic mood, whereas a Sardinian may perform it as part of feeling offended and aggressive.

It is important to appreciate that, for the most part, these influences of culture on various aspects of mood do not entail the conscious endorsement of specific rules and values, and/or conscious control of feelings and behaviour on the part of individuals. Rather, cultural norms and values mould people’s behaviour and feelings gradually, largely via widespread mechanisms of facial and postural mimicry that also mediate mood contagion (Barsade 2002; Hatfield et al. 2009). Through these mechanisms, our environment inscribes its cultural norms into our moods, leading us to acquire culture-specific ways of feeling and behaving that can be regarded as part of our *habitus,* or set of incorporated social practices (e.g., Mauss 1935; Bourdieu 1980; Connerton 1989). These include spontaneous ways not just of moving and expressing our affective states, but also of responding affectively and regulating how we feel.

### Environmental Scaffolds

I now want to introduce a concept that has recently garnered increasing attention in the philosophy of cognitive science. This is the concept of *scaffolding,* which a number of philosophers have used to emphasize that our cognitive capacities are often supported and enhanced by our interactions with concrete aspects of the environment (broadly, objects and people). By applying this concept to the case of moods, we can illustrate another sense in which moods are (non-trivially) situated in the world.^6^

Inherent in the notion of scaffolded cognition is the idea that certain cognitive feats cannot be achieved by the organism alone, but need to be supported and structured by the environment. Developmental psychologists influenced by Vygotsky introduced the term “scaffolding” to refer specifically to the role of teachers and caregivers in helping infants learn new cognitive skills that are within their “zone of proximal development” (Wood et al. 1976). More recently, philosophers have appropriated the term to emphasize that our cognitive capacities (perception memory, reasoning, learning) are supported and vastly enhanced by the many tools we build and use—such as binoculars, calculators, maps, clocks, personal computers and wearable technologies (e.g., Clark 2003; Sterelny 2010). These accounts in particular also emphasize the *reciprocal influences* occurring between organism and environment: organisms build material scaffolds that enhance their cognitive capacities, which lead to new scaffolding practices, and so on. Sterelny (2010) thus also regards cognition-scaffolding processes as instances of “niche construction”. In biology, this term refers to an organism’s construction or modification of its environment, and subsequent adaptation to it, in ways that change the organism’s behaviour and improve its survival and fitness (think of the beaver’s dam). For Sterelny, our widespread practices of structuring the environment and building tools to improve our cognitive capacities are best characterized as instances of *epistemic* niche construction (see also Clark 2005). Finally, philosophers have pointed out that scaffolds exert their influence at different timescales. Griffiths and Scarantino (2009) claim that emotions are both diachronically and synchronically scaffolded: the environment provides long-lasting structures that mould emotions over the long term, as well as more temporary supports. Wilson and Clark (2009) similarly distinguish between one-off transient couplings of organism and environment that temporarily enhance a cognitive capacity (as when one uses a calculator to perform a complex mathematical operation) from more robust and durable couplings where the environment scaffolds mental capacities over one’s lifetime or even over generations (think of the introduction of written language in human cultural history).

All these ideas apply to moods as well, and indeed reflecting on the scaffolded nature of our moods brings to the fore something that has not been emphasized much so far (quite the contrary)—namely, the important fact that moods are not simply *passive* responses to environmental stimuli, but that *we act* on our surroundings to influence our moods, scaffolding them and creating short- and long-term *affective niches* to which we adapt, and in which we achieve moods that would otherwise not be possible. This point is worth making for at least two reasons. First, philosophers of cognitive science (including of embodied and situated cognition) have primarily analysed affect-less cognitive/epistemic scaffolding practices, with little or no consideration for affectivity. Second, moods in the philosophy of emotion have been regarded as especially resistant to reasons (more so than emotions; see, e.g., Lormand 1985; Sizer 2000). Relatedly, it is tempting to regard moods as essentially passive and largely outside our control. Pointing out that they are environmentally scaffolded serves to show that there are elements of activity in moods as well: moods are not just affective states that happen to us and that take us over for no apparent reason, but to some extent we can also actively bring about our moods. In fact, we can regard the act of scaffolding our moods as involving both active and passive elements: we actively manipulate the environment so that we can be passively influenced by it.

There are innumerable examples of both synchronic and diachronic mood-scaffolding practices. Perhaps most obviously, consider the widespread use we make of ingestible chemicals: we take caffeine to keep alert and energized, alcohol to wind down and relax, chocolate to feel comforted, Prozac to treat depression, and so on (just to mention legal drugs). Yet, taking drugs is not the only or even most common way we rely on the world for the purpose of altering our moods. I, for one, often choose clothes and accessories in the morning to influence how I feel—selecting for example bright colours or a citrus-scented perfume to cheer me up. We also know from work in sociology that another common accessory, the handbag, plays an important role in the life of many people who rely on this object’s looks and feel to regulate their mood—for example by choosing a certain colour to brighten up their day, or a certain texture and shape for comfort and reassurance (see Kaufmann 2011; Colombetti 2015). The contents of the handbag can also rekindle fond memories (in the case of photographs and old cinema tickets, for example), or foster a sense of self-assurance and independence (in the case of diaries and organizers). Many people also carefully choose furniture and decorate their homes to achieve aesthetic effects that influence their mood; it is not uncommon to see affective language explicitly used on the front covers of design and home-decoration magazines, such as “comfort”, “heart” and “love”. The very notion of “home” has connotations of familiarity and safety, and many treat it as a space they can design and personalize to achieve a variety of effects on their mood. The home is perhaps best regarded as a large “container niche” with a number of constituting and often overlapping affective niches inside it: the kitchen is where one can prepare comforting food, the living room where one can lounge and relax, and so on. Listening to music is, of course, another widespread mood-scaffolding activity that takes many forms—from listening to personal portable music players, to putting music on at home to create a certain atmosphere for a dinner party, to going to concerts (see DeNora 2000 and Krueger 2014 for the view that music “entrains” our affective states). We can add to this list the use that we also make of people and social events to influence our moods—from visiting friends and relatives to going to parties, support groups, or sport events (see Colombetti and Krueger 2015 for a more extensive discussion of material and intersubjective affective scaffolds).

All these scaffolding activities can be regarded as world-involving mood-regulatory practices. In his extensive discussion of mood regulation, the psychologist Robert Thayer (1996, 2001) emphasizes that people resort to all sorts of strategies to modify their moods—such as engaging in some cognitive activity, exercising, eating sugary foods, shopping and talking to others. In his view, we constantly (and more or less consciously) change our relation to the world, trying to maintain positive mood states and to avoid negative ones. Our self-regulatory strategies aim primarily at increasing energy and/or lowering tension, to achieve an optimal positive mood state Thayer calls “calm energy”. Importantly, most of these strategies clearly involve utilizing some part of the environment. In this sense, the expression “mood scaffolding” can certainly be regarded as another term for (at least some forms of) mood regulation,^7^ and Thayer’s overall account is compatible with the present view that moods are both embodied and situated. Note, however, that most psychologists today approach self-regulation from a cognitivist perspective, studying and highlighting self-regulatory strategies such as re-appraising one’s situation and/or relevant stimuli, and/or redeploying attention to something else (e.g., Gross 2014). This perspective tends to characterize self-regulation as something that happens solely or primarily “inside the head” (in particular within those parts of the brain traditionally considered loci of cognitive control, such as the prefrontal cortex). It is true that Gross also mentions “situation selection” and “situation modification” as regulatory strategies that involve physically removing oneself from a certain situation, or modifying it to remove its consequences. Yet he does not say much about these situated practices—which are, arguably, significantly different from the cognitive-internal ones to which he pays more attention. Some psychologists, in fact (see Koole and Veenstra 2015), have recently pointed out that Gross’s influential model of self-regulation places too much emphasis on the activation of internal explicit and verbalizable goals, and have called for a more embodied and situated approach that also takes into account the formation of habitual patterns of reliance on the environment to achieve specific affective states.

In addition, the notion of mood scaffolding adds something important to the one of mood regulation—or so I want to suggest. Shopping, cooking, eating, etc. are arguably not just “distractions” (Thayer 1996, p. 116) aimed at increasing our energy levels and/or lower our tension (although these may well be primary motivating aims). The *specific material features* of the environment with we choose to interact, I maintain, also play a role in mood regulation, contributing unique qualities that help us achieve specific moods. For example, I may choose to bake pizza—instead of, say, drink alcohol—not just to lower my level of tension, but because of the specific sensuous aspects of that activity (the feel of kneading the dough, the smell of the pizza), which are missing from the activity of drinking alcohol (of course, other people, or myself in other circumstances, may prefer the latter activity, given its own specific sensuous features). In other words, the notion of “scaffolded moods” opens the door to the recognition that some of the reasons we rely on the world to regulate our moods is that the world has unique material qualities which allow us to achieve specific moods that would otherwise be outside our reach. Likewise, the related notion of “affective niche construction” I introduced earlier describes the active establishing of specific organism-world relations that make particular moods possible in virtue of distinctive properties of the niche. In sum, the notions of affective scaffolding and niche construction can help us characterize practices of mood regulation more precisely, emphasizing the role of distinctive material features of the world in determining the specific moods we enter into.

### Incorporations

Mood scaffolds, as we have just seen, are widespread and come in all sorts of varieties. In this subsection I turn my attention to a limiting case of the phenomenon of mood scaffolding, which occurs when the parts of the world that scaffold one’s moods are experienced as parts of oneself. Drawing on phenomenological philosophy, I call this phenomenon *incorporation*.

There are at least two ways in which parts of the world can be experienced as part of oneself. William James identified one of them when he wrote that

*a man's Self is the sum total of all that he* CAN *call his*, not only his body and his psychic powers, but his clothes and his house, his wife and children, his ancestors and friends, his reputation and works, his lands, and yacht and bank account. All these things give him the same emotions. If they wax and prosper, he feels triumphant; if they dwindle and die away, he feels cast down, not necessarily in the same degree for each thing, but in much the same way for all. (James 1890, p. 291-292)

I interpret this passage as claiming that we come to regard certain objects and people *as part of who we are, in particular of our social identity*.^8^ The passage also suggests that the sign that this is the case is that we feel toward these objects and people the same way we feel toward ourselves (our body and mind). It is likely, however, that the affective attachments we develop toward certain objects and people in the course of our lives also contribute to making them into particularly significant items that we come to regard as necessary for all sorts of activities and relationships without which we would not be who we are. In any case, more recently James’s proposal that we regard our possessions as constitutive of our identity has garnered empirical support from the fields of socioeconomic psychology and consumption studies. Prelinger (1959), for example, asked subjects to sort items depending on their relation to the self, and found that subjects listed “possessions and productions” as mostly related to the self, just after body parts, psychological processes and personal identifying characteristics (see also Csikszentmihalyi and Rochberg-Halton 1981). Belk (1988), in his defence of the idea that possessions *extend* the self, mentions various other sources of empirical evidence for this claim—such as evidence that people who lose cherished possessions (e.g., in an earthquake or burglary) feel a diminishment of their selfhood (which is also why in certain institutions, such as prisons or concentration camps, it is common practice to take personal items away from inmates). The practice of burying the dead with their possessions can also be regarded as a manifestation of the idea that possessions are part of one’s identity.

As James (1890) already noted, this kind of evidence could be interpreted in mere utilitarian or instrumental terms: we value our possessions because they enable us to do a variety of things; losing them brings us sorrow or even depression because we lose the ability to do such things. This instrumental interpretation, however, does not explain why losing certain objects comes with experiences of deep loss, personal attack and violation and, to quote James again, “a sense of the shrinkage of our personality, a partial conversion of ourselves to nothingness” (1890, p. 293). Of course, losing something useful or that we value can bring about a negative mood—as when we lose an umbrella, a glove, or money. But to lose something that we experience as part of our history and identity arguably affects us more deeply—just imagine losing your favourite books in a fire, a gift from your best friend, or a piece of jewellery that belonged to a beloved grandparent (not to mention the tragic experience of losing a longstanding partner). Losing such things can lead to a depressed mood that includes a deeper sense of loss, a feeling of having somehow been severed and diminished. Interestingly, we are often not aware, before we lose something or someone, of the role it plays in our sense of identity and in our everyday affective condition, including moods—which supports the point that the way we ordinarily feel is due, more than we think, to certain things and people. It seems thus possible to say that our everyday moods of, e.g., self-confidence, assurance and safety, depend on the tacit awareness of certain things and people as part of our identity.

A second way in which parts of the world can be experienced as part of oneself is by being *incorporated into a person’s pre-reflective awareness of her or his body*. Indeed, the notion of incorporation in phenomenological philosophy refers primarily to this case. According to Merleau-Ponty (1945), for example, the blind person experiences her cane not as an external physical object, but as something that, like the person’s lived body (e.g., her hand), makes the experience of the world possible. The cane, in Merleau-Ponty’s terminology, is incorporated or taken into the person’s “body schema”, that is, it becomes part of the habitual bodily structures that enable the world to show up in experience the way it does. The incorporated cane is experienced not as an object, but as part of one’s subjective, bodily self. Like the person’s body, the cane is lived pre-reflectively or tacitly, as that through which something else is experienced as an object. Consider also the example of writing on a blackboard with a piece of chalk (Ihde 1979): the piece of chalk is experienced as that through which the blackboard shows up as hard and smooth, similarly to when the person smears the blackboard with the tip of her fingers.

My suggestion here is that moods can include the experience that parts of the world are part of one’s pre-reflective bodily self, where these parts also play a crucial role in making the moods in question possible. Perhaps the best way to illustrate this possibility is with the example of an instrumental musician who plays to express and give form to a mood—of, say, longing or sadness (see also Colombetti 2016). In this case, the musician’s attention is on the music and his feelings, and he experiences the instrument not as an external physical object, but as *that through which* he can articulate a mood. We can draw a parallel here with a dancer who dances to express and articulate a certain mood. She clearly does not experience her body as an object, but as a subject of expression. Likewise, I want to suggest, the instrumental musician experiences his instrument as part of his subjectively lived, mood-expressing body; the instrument has been taken into the musician’s experience of his body as a performing, mood-articulating subject of experience.

Consider also the example of a fashion-conscious person, who always carefully chooses how to dress in social occasions. Whenever she believes she is appropriately dressed, she feels confident, interacts smoothly with others, is cheerful and funny; on the other hand, when she does not wear what she thinks is appropriate, she feels upset and out of place. In such a case, we can say that the person’s self-confident and cheerful mood includes the incorporation of her clothes into, this time, her “body image”—that is, the image she has of how others regard her body. Again, this experience is best characterized as pre-reflective or tacit: as the person interacts cheerfully with others at a party, for example, she does not pay attention to her smart suit; rather, the suit and its smart character are present in the person’s experience implicitly, in the background, as something enabling her to be confident and cheerful. Likewise, the confident bodybuilder, say, experiences his body as strong and attractive to others in a pre-reflective way, as something that enables him confidently to inhabit the world and his interactions with other people. The case of the fashion-conscious person is analogous—with the only difference that, here, it is the clothed body that is experienced as confidence-enabling.

## Conclusion

I began my discussion arguing against the view, widespread among affective neuroscientists, that brain activity constitutes *the* physical basis or “core machinery” of moods, and that affective states are “in the brain”. I have proposed instead to regard moods as *embodied*. According to this view, the physical basis of moods includes not only brain processes, but also bodily activity interacting with the latter. As we have seen, the brain is deeply interrelated with the rest of the organism, and we know now that substances synthesized locally in the endocrine and immune systems influence moods such as depression and stress-related conditions. Although more empirical data are needed, there is no reason to think that ordinary moods (grumpiness, elation, ennui, and so on) should not be similarly underpinned by neurochemical processes that criss-cross the boundary between the brain and the rest of the organism.

Second, I have emphasized that moods are also *situated* in the world. Although this claim is fairly trivial, it has not been much discussed in the philosophy of situated cognition, which so far has highlighted primarily the situated character of our (non-affective) cognitive capacities. To fill this gap, I have argued that moods are situated, not just in the trivial sense that they are influenced by events and situations in the world, but in the sense that they are complexly interrelated with the world, in at least three different ways: i) the world provides cultural values and norms that influence how we feel and behave in mood, including how we regulate such feelings and behaviours; ii) the world is also something that we actively manipulate and modify to construct affective niches in which we can undergo moods that would otherwise be out of our un-aided organism’s reach; and, finally, iii) sometimes our moods include experiencing aspects of the world as part of ourselves—either of our identity, or of our pre-reflective bodily self-awareness.

Taken together, these considerations indicate that to fully understand human moods, we need to consider not just the neurochemistry of the brain but also of other parts of the organism, as well as the broader context, or rather contexts, in which the organism is immersed. Given the unceasing and pervasive character of the interactions among brain, body and world, it is perhaps easy to take them for granted, and to focus on a few brain systems only, coming to think they constitute *the* physical basis or core machinery of moods. But it is precisely because of this unceasing and pervasive interactivity that our moods are not confined to our heads, and that we need to take seriously the contributions of the body and the world to advance our understanding of these fascinating phenomena.

## Footnotes

1 Expressions such as “the neural basis” and “the brain basis” of emotions and moods are widespread in the neuroscientific literature and its popularization. Articles that characterize depression as brain-based are, for example, Castrén (2005), Drevets et al. (2008), and Pandya et al. (2012). The latter titled their article “Where in the brain is depression?”; similarly, Wehrenberg and Prinz (2007) titled their book on the neuroscience of anxiety *The Anxious Brain*. An anonymous referee (a neuroscientist) for this paper reiterated in his/her review that affective states are “in the brain”, whereas bodily processes have only a “modulatory” function. The sceptical reader can also verify this tendency by doing an internet search on PubMed and Google.

2 I do not think that only humans can have moods (see Colombetti 2014, chapter 1), but for the purposes of this paper I will leave this point aside.

3 Neurotransmitters and neuromodulators are chemical messengers (peptides) that affect the activity of neurons. Neurotransmitters are released by presynaptic neurons, and very rapidly influence nearby postsynaptic neurons. Generally, neuromodulators have more complex effects than neurotransmitters; for example, they can modify postsynaptic responses to specific neurotransmitters, as well as the synthesis, release or metabolism of neurotransmitters in presynaptic neurons. The influence of neuromodulators usually unfolds at longer time-scales, and induces changes in enzymatic activity, as well as in protein synthesis via influences on DNA transcription. The difference between neurotransmitters and neuromodulators, however, is not always clear-cut, given that they are often synthetized and released together by the same presynaptic neurons (Widmaier et al. 2014).

4 The argument would become more complicated if we also considered recent findings of the influence of gut microbiota on behaviour, mood and cognition (see Jenkins et al. 2016). These findings challenge traditional conceptions of the organism and complicate the notion of embodiment. I shall leave the discussion of this (fascinating) point to another occasion.

5 Psychologists also talk of “emotion regulation” and “affect regulation” (or self-regulation), often however without explaining whether these expressions are meant to refer to different phenomena. This lack of clarity is partially due to the fact that many psychologists do not distinguish neatly between emotions and moods (see Fox 2008, chapter 2) and think that “affect” (positive or negative feelings) characterizes both. In this paper, by “mood self-regulation” I refer specifically to the regulation of one’s own moods rather than emotions.

6 As I explain below, the notion of scaffolding has been applied primarily to non-affective cognitive capacities. One exception are Griffiths and Scarantino (2009), who use this notion to argue that emotions are situated. Their discussion focuses primarily on the environmental support offered by other people (see also Colombetti and Krueger 2015).

7 Thanks to the editors for pointing this out.

8 One can also read the passage as saying that certain objects and people *are*, objectively, part of the self.
